# A REST derived gene signature stratifies glioblastomas into chemotherapy resistant and responsive disease

**DOI:** 10.1186/1471-2164-13-686

**Published:** 2012-12-07

**Authors:** Matthew P Wagoner, Avtar Roopra

**Affiliations:** 1 Department of Neuroscience, University of Wisconsin at Madison, Madison, USA; 2 Molecular and Cellular Pharmacology Graduate Program, University of Wisconsin at Madison, Madison, USA; 3 Medical Science Center, Room 5675, University of Wisconsin at Madison, 1300 University Ave, Madison, WI, 53706, USA

**Keywords:** REST, RE1, NRSE, NRSF, Glioblastoma, Patient data, Chemotherapy, Bioinformatics

## Abstract

**Background:**

Glioblastomas are the most common central nervous system neoplasia in adults, with 9,000 cases in the US annually. Glioblastoma multiformae, the most aggressive glioma subtype, has an 18% one-year survival rate, and 3% two year survival rate. Recent work has highlighted the role of the transcription factor RE1 Silencing Transcription Factor, REST in glioblastoma but how REST function correlates with disease outcome has not been described.

**Method:**

Using a bioinformatic approach and mining of publicly available microarray datasets, we describe an aggressive subtype of gliomas defined by a gene signature derived from REST. Using this REST gene signature we predict that REST function is enhanced in advanced glioblastoma. We compare disease outcomes between tumors based on REST status and treatment regimen, and describe downstream targets of REST that may contribute to the decreased benefits observed with high dose chemotherapy in REM tumors.

**Results:**

We present human data showing that patients with “REST Enhanced Malignancies” (REM) tumors present with a shorter disease free survival compared to non-REM gliomas. Importantly, REM tumors are refractory to multiple rounds of chemotherapy and patients fail to respond to this line of treatment.

**Conclusions:**

This report is the first to describe a REST gene signature that predicts response to multiple rounds of chemotherapy, the mainline therapy for this disease. The REST gene signature may have important clinical implications for the treatment of glioblastoma.

## Background

RE1-silencing transcription factor (REST) is a transcriptional repressor that regulates the expression of approximately 2,000 neuronal genes in neural and non-neural tissues, including embryonic and neural stem cells (NSC) [1-4]. In development, loss of REST function is an integral step in NSC differentiation, and inappropriate maintenance of REST function has been found to prevent differentiation of NSC into neurons [4-6]. In neoplasia, heightened REST function in medulloblastoma tumor cells contributes to their tumorigenicity in mouse models of the disease, in part by preventing their differentiation [7,8]. Accordingly, many human medulloblastoma tumors show significantly higher REST protein levels than adjacent normal brain tissue. Increased REST levels also correlated with lower overall and event-free survival [9].

Glioblastomas are the most common central nervous system neoplasia in adults, with 9,000 cases in the US annually. Regardless of whether gliomas arise from astrocytes (astrocytomas) or oligodendrocytes (oligodendromas), they carry with them a uniformly poor prognosis. Glioblastoma multiformae, the most aggressive glioma subtype, has an 18% one-year survival rate, and 3% two-year survival rate [10]. Recently, a role for REST was suggested in glioma with REST up-regulation able to drive cell proliferation and suppress differentiation [11,12]. Elegant work from Conti *et al* and Kamal *et al* showed increased REST protein in glioblastoma samples versus control brain tissue and knockdown of REST in glioblastoma cell lines reduced proliferation rate and promoted differentiation. However, how REST levels corresponded to patient outcome was not described. In this work, we will describe an aggressive subtype of gliomas with enhanced REST function, termed REST Enhanced Malignancies (REM). We compare disease outcomes between tumors based on REST status and treatment regimen, and describe downstream targets of REST that may contribute to the decreased benefits observed with high dose chemotherapy in REM tumors.

## Results and discussion

**Figure 1:**
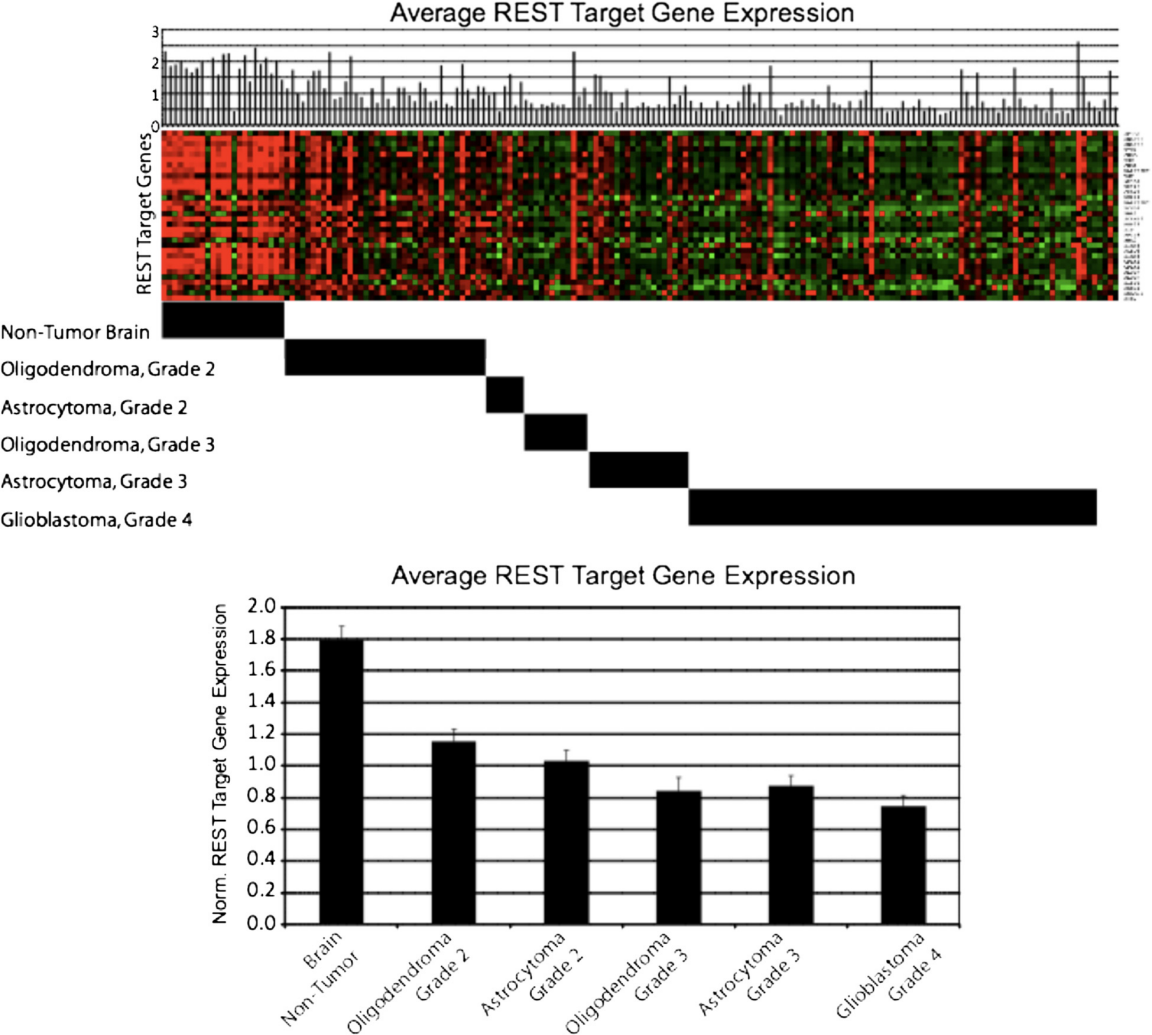
**Expression of REST signature genes in glioma tumors and non-neoplastic brain tissue.** Expression REST signature genes (y-axis) are compared between 176 glioma tumors and non-neoplastic brain tissues (x-axis) from NCBI dataset GSE4290. Non-neoplastic brain tissue shows significantly greater REST target gene expression than many of the oligodendroma and astrocytomas. Average expression of normalized REST target genes is displayed below as mean +/- standard error. Oligodendromas and astrocytomas both show decreased REST target gene expression with increasing tumor grade. All heatmaps and tumor analyses were performed using MultiExperiment Viewer v4.6.

**Figure 2:**
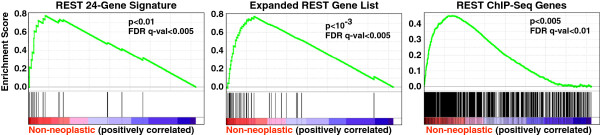
**GSEA comparing REST target gene expression in glioma tumors and non-neoplastic brain.** Gene set enrichment analysis results are shown comparing gene expression between 176 glioma tumors (of varying grade and type) and non-neoplastic brain (NCBI Dataset number GSE4290) over 3 independent lists of REST target genes. These results suggest that when compared to normal brain tissue, the glioma tumors in this dataset show a robust down-regulation of REST target genes. The FDR q-value and nominal p-value for each genelist suggest that such an enrichment of REST target gene expression in non-neoplastic tumors is unlikely to occur by random chance. GSEA was performed using 1,000 permutations utilizing a weighted enrichment statistic and a Signal2Noise metric for ranking genes. Genes with multiple probes were collapsed into a single gene using the max-probe value. The REST signature genelist is comprised of 24 genes that all contain REST binding sites and responded to REST knockdown with at least a two-fold up-regulation of target mRNA in three different cell lines, as previously described (Wagoner *et al.* 2010). The expanded REST genelist contains an additional 30 REST target genes that are up-regulated at least two-fold with loss of REST function in two of three cell lines. The 971 REST ChIP-Seq genes were determined by ChIP-Seq analysis from Jurkat t-cells by Johnson *et al*[3].

To evaluate REST function in gliomas we utilized a 24-gene REST gene signature which has been demonstrated to be a reliable reporter of REST function in tumors [13]. Using this gene signature, we evaluated REST function in a dataset of 176 neural tissue samples, including non-neoplastic brain tissue as well as oligodendromas and astrocytomas of varying grades (Figure 1) [14]. Both the oligodendromas and astrocytomas showed a statistically significant decrease in REST target gene expression with respect to their non-neoplastic counterparts. Intriguingly, both glioma subtypes showed significant decreases in mean REST target gene expression with increasing tumor grade, suggesting that heightened REST function may be associated with more aggressive disease. We also evaluated REST function in these tumors using gene set enrichment analysis (GSEA), which measures the relative enrichment of a given geneset in one tumor population over another in a statistically rigorous manner [15]. Using GSEA, we compared REST function in gliomas and non-neoplastic brain tissue using 3 independent lists of REST target genes (Figure 2). GSEA found that the genes in the REST 24-gene signature were indeed down-regulated in a statistically significant manner in a subset of gliomas (p<0.05, FDR q-val<0.05). A second gene list examining 30 REST target genes not present in the 24-gene signature that were induced at least 2-fold in at least 2 of three cell lines tested in Wagoner *et al*[13] was also strongly under-expressed in the same subset of gliomas. This suggests that enhanced REST is not limited to a small set of REST target genes (p<0.001, FDR q-val<0.01). Finally, GSEA was performed based on a geneset comprised of over 800 REST target genes identified in Jurkat T-cells as REST targets by ChIP-Seq after removal of the 24-gene signature (p<0.001, FDR q-val<0.01) [3]. This analysis confirms the statistically significant down-regulation of REST target genes in gliomas with respect to non-neoplastic tissue, suggesting an increase in REST function in the tumors.

**Figure 3:**
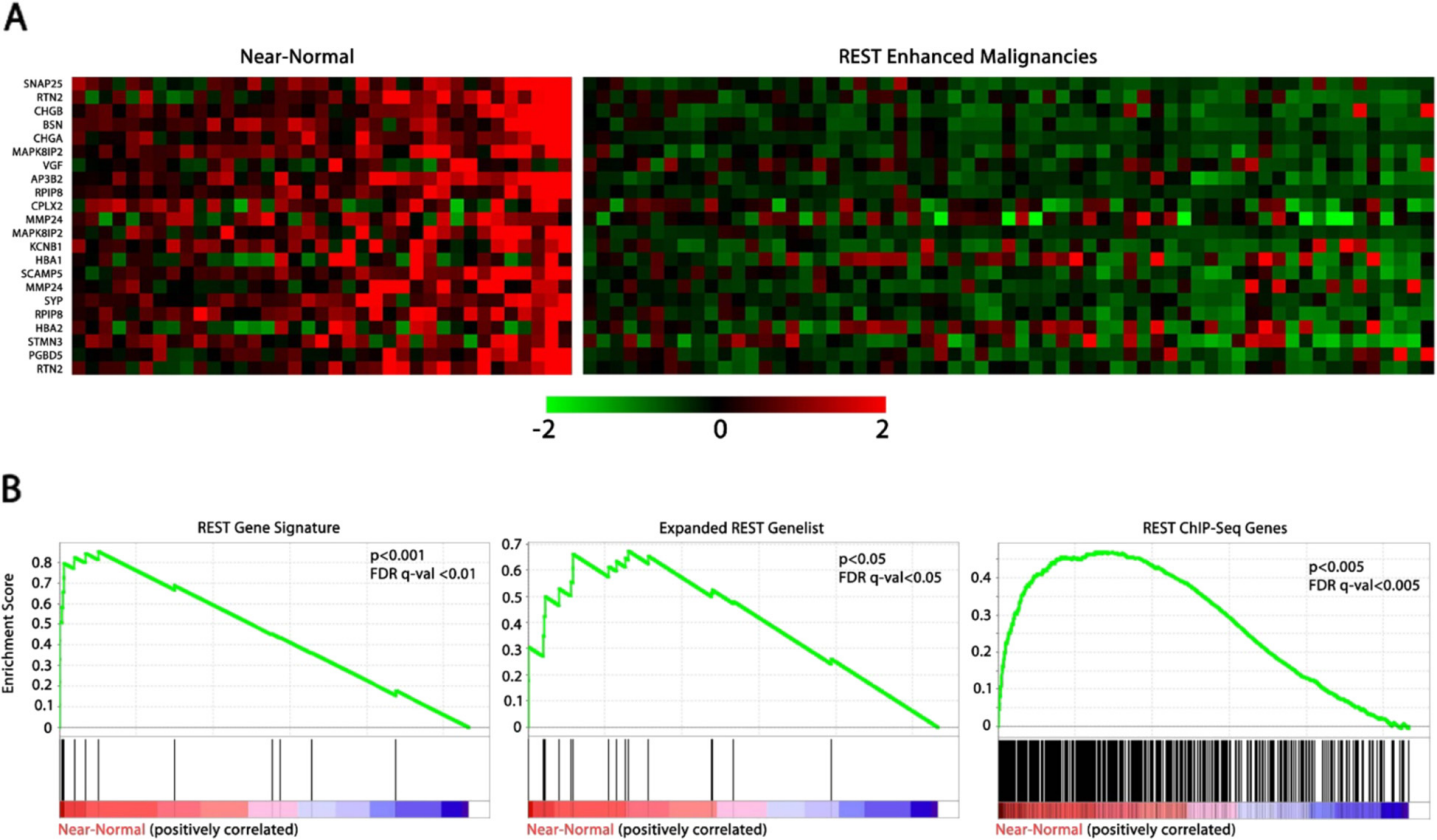
**High grade gliomas have varying levels of REST target gene expression.** Grades III and IV gliomas from NCBI Dataset GSE4271 were broken into groups expressing high and low levels of REST signature genes (termed near-normal and REM tumors, respectively) using self-organizing maps and a euclidian distance metric. (**A**) Heatmap of REST signature gene expression for the 100 high-grade glioma tumors. (**B**) GSEA was used to determine if the differences in expression of REST target genes between REM and near-normal tumors are statistically significant. These results suggest that REM tumors indeed show significantly depleted expression of a wide range of REST target genes. GSEA parameters and genesets were utilized as in Figure 2.

Intriguingly, the increased REST function observed in gliomas was not uniform across all tumors, with some tumors expressing REST target genes near the levels observed in non-neoplastic tissue. To determine whether the intertumoral variation of REST function is significant, we ranked tumors by expression of the 24-gene signature and then divided gliomas into groups of high and low expression of REST signature genes. Tumors with low level expression of genes in the REST signature were termed REST enhanced malignancies (REM), and those with expression levels of REST target genes at or near that of normal, non-neoplastic tissue were termed “near-normal” tumors (Figure 3A). Using the above three independent REST gene lists, GSEA found statistically significant decreases in REST target gene mRNA levels, suggesting that a significant population of these high grade gliomas have heightened REST function (Figure 3B).

**Figure 4:**
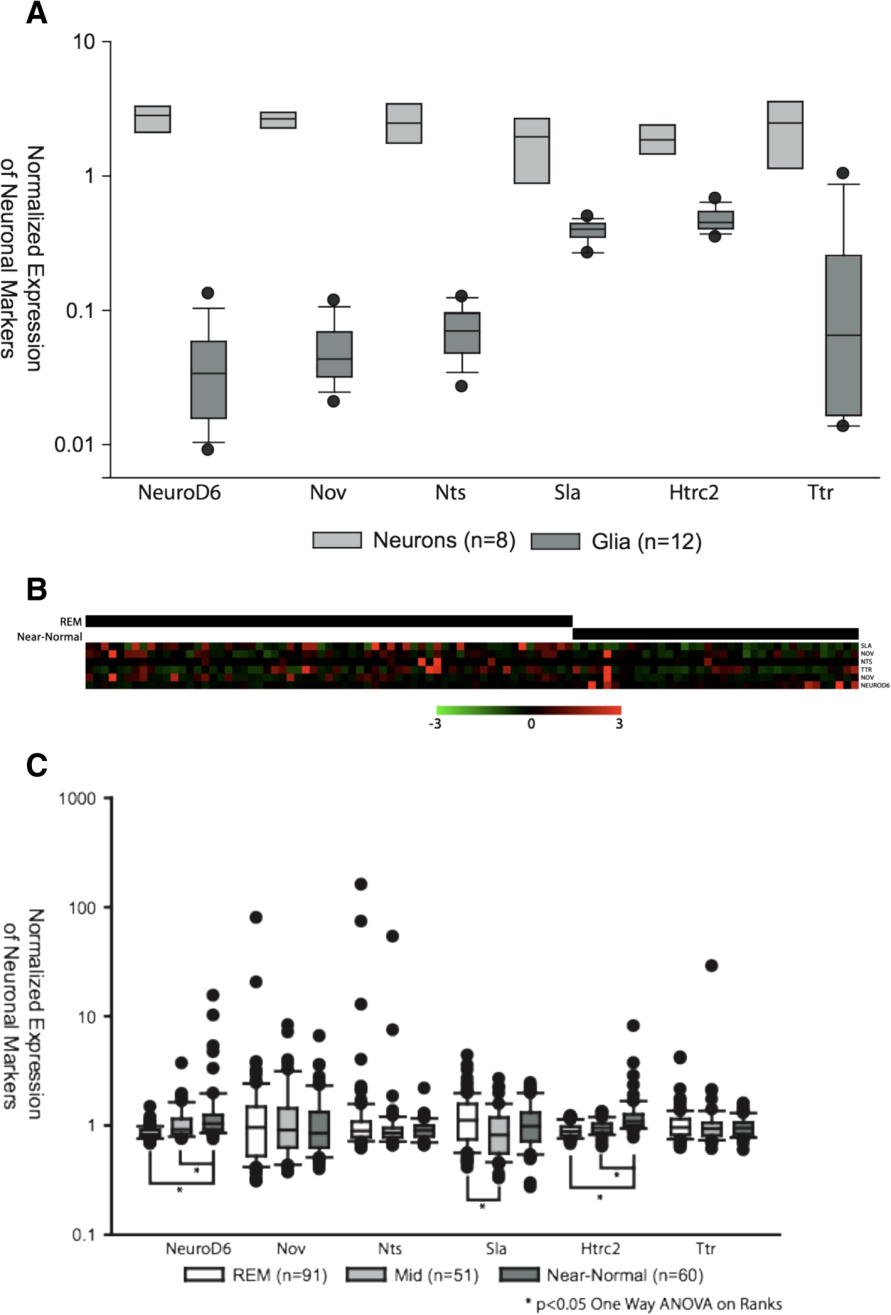
**Neuronal markers do not segregate with REM or near-normal tumors.** (**A**) Box plot of neuronal markers derived from dataset GSE9566, which profiles genes expressed in pure populations of astrocytes, oligodendrocytes and neurons (Cahoy, Emery *et al.*[2008]). Genes that were selectively expressed in neurons (p<10^-15^) were filtered out if they contained a consensus REST binding site within 30kb of the gene, or if REST had been published to bind the gene in ChIP-ChIP or ChIP-Seq experiments [3,17]. Top, middle, and bottom lines of the box plot represent 75th, 50th and 25th percentile samples, respectively. The top and bottom whiskers represent 90th and 10th percentile samples, respectively. (**B**) Heatmap showing expression of neuronal markers in REM and near-normal tumors from gliomas in dataset GSE4271. Neither REM nor near-normal tumors show significant enrichment for non-REST associated neuronal markers. (**C**) Neither REM, Mid-, or Near Normal subtypes show a significant concerted increase in all non-REST target neuronal markers suggesting equal levels of neuronal involvement in the tumor.

Given that many REST target genes are highly expressed in mature neurons, one possible explanation for the elevated REST function observed in REM tumors could be that those tumors have low levels of neuronal involvement or “neuronal contamination”. To determine if higher levels of neurons are present in near-normal glioma tumor samples with respect to REM tumors, we first had to identify genes selectively expressed in neurons that are not likely REST target genes. These genes were selected from a gene expression dataset comparing fluorescently sorted neurons, astrocytes, and glia from the murine CNS [16]. First, we identified those genes that are most highly and selectively expressed in neurons. Then we filtered out any genes that had been identified as a potential REST target in published ChIP-ChIP or ChIP-Seq experiments, or contained a consensus 21bp REST binding element [3,17]. Figure 4A validates the resulting 6 genes that are not REST targets as neuron specific.

Evaluation of neuronal non-REST target genes found that there was no concerted up-regulation of all neuronal non-REST markers in either REM or near-normal tumors. Similarly, Vascular endothelial markers VEGFR and VE-Cadherin were not enriched in REM tumors (data not shown) Thus we conclude that different levels of neuronal or vascular involvement between tumors are not responsible for the observed variation in REST function (Figure 4B and C).

**Figure 5:**
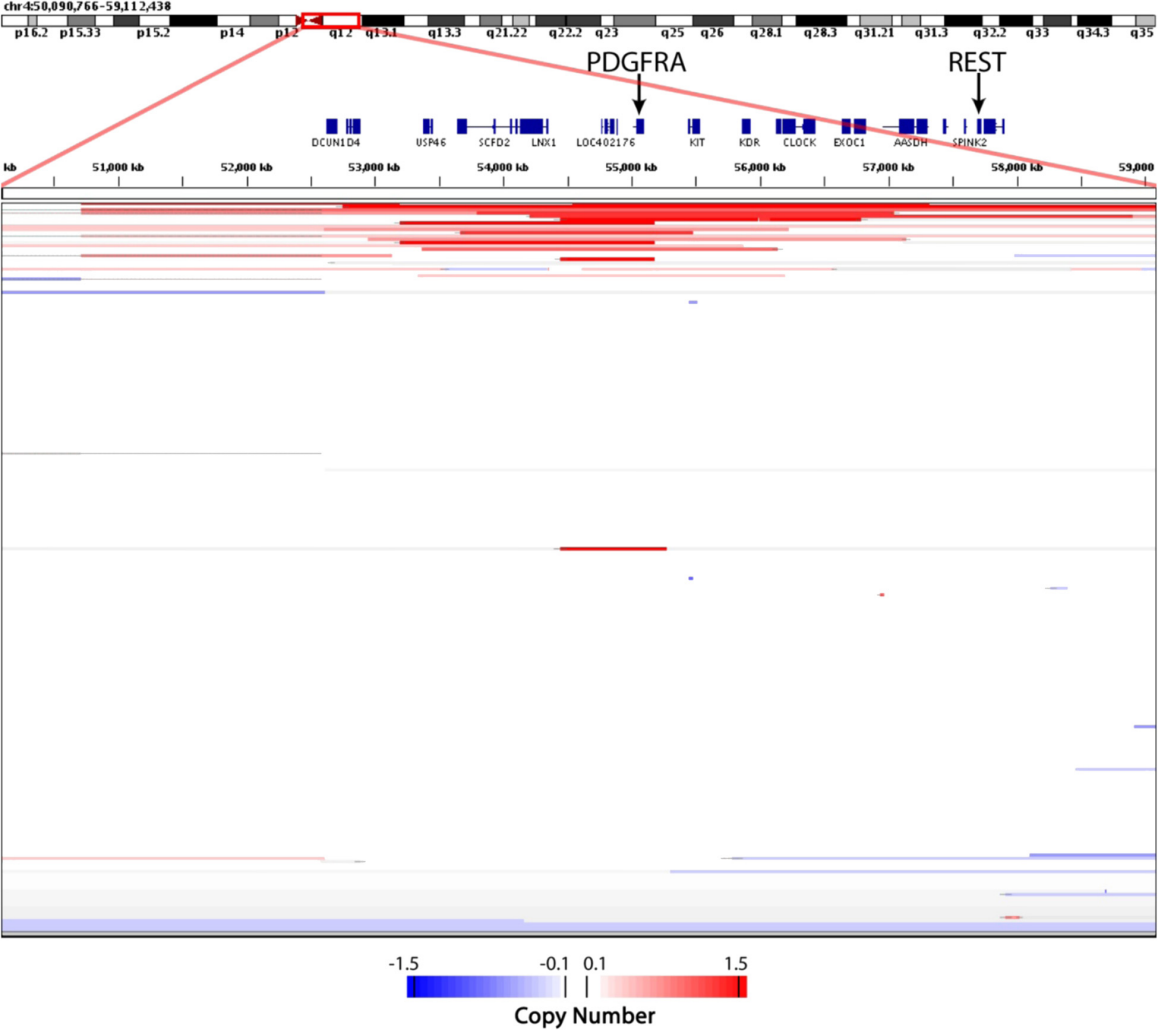
**Glioma Copy Number Variation In Chromosome 4q12.** Segmentation analysis depicts 4q12 (x-axis) amplification and deletion events representing 141 glioma tumors (y-axis). Exons for known genes are highlighted as blue boxes, with introns depicted as blue lines between boxes. The glioma oncogene PDGFRA is the focus of 4q12 amplification, consistent with what is normally seen in the literature.

In trying to determine a possible molecular cause for the enhanced REST function, we first examined the likelihood that it was due to altered levels of REST expression. The gene encoding REST is located in chromosome 4q12, a region of frequent focal amplification in aggressive glioblastomas. Analysis of publicly available copy number data for 141 glioma tumors, however, found that the amplification of 4q12 was centered around PDGFRA, a known glioma oncogene (Figure 5) [18] and not around REST. In these samples, PDGFRA was the target of frequent focal amplification, often without the coincident amplification of REST, which is located 2,500kb downstream (Figure 5). We next asked if the enhanced REST function was due to increased REST mRNA. Although Conti *et al* found a two- to five-fold increase in REST mRNA between normal and malignant tissue, we found no increase in REST mRNA levels in glioma tumors, or correlation between REST mRNA levels and REST target gene expression in the datasets we examined (data not shown). The basis for this discrepancy is not clear. However given the poor correlation between REST transcript levels and protein levels in gliomas [12], it is possible that our meta-analysis picks up a different, though perhaps overlapping group of tumors than those described in Conti *et al*[11]. Thus the signature approach would identify glioblatomas that had increased REST function or protein levels in the absence of changes in mRNA levels.

**Figure 6:**
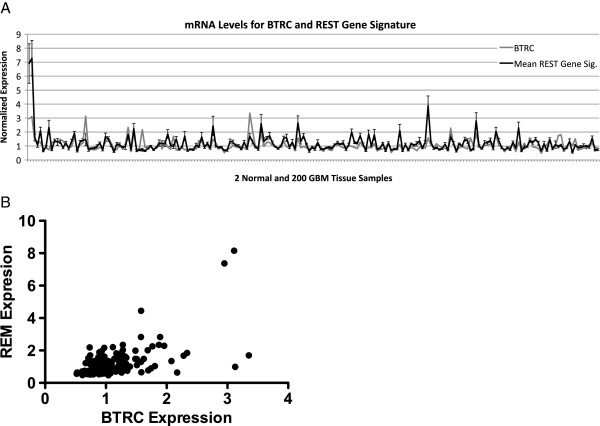
**Correlation of BTRC mRNA and REST signature gene levels.** (**A**) Mean normalized REST signature gene and BTRC mRNA levels are plotted for 2 non-neoplastic and 200 GBM tissue samples from the cancer genome atlas’ (TCGA) unified and scaled gene expression dataset [21]. A strong correlation between REST function and BTRC mRNA levels is confirmed to be statistically significant using the Kendall’s rank correlation test p-value (p<10^-17^). (**B**) Scatter plot of data in (**A**). Statistical analyses were performed using the Mstat software package (http://www.mcardle.wisc.edu/mstat/).

Given that heightened REST function is not due to enhanced REST copy number or gene expression, we investigated possible post-transcriptional mechanisms of regulating REST function. In development, REST function is modulated in multiple ways. In neuronal stem cells, loss of REST function is necessary for differentiation into neurons [19]. But before REST is lost at the mRNA level in differentiating neural stem cells, its function is ablated through the expression of the the F-box protein β-TrCP, an E3 ubiquitin ligase [5,20]. β -TrCP ubiquitinates REST, resulting in its degradation and the de-repression of REST target genes. We posited that β-TrCP levels maybe similarly regulating REST function in these gliomas. Analysis of gene expression levels in normal and neoplastic brain tissues revealed a striking correlation between the expression of REST target genes and β -TrCP (Figure 6) (p<10^-17^) [21]. This correlation is consistent with the hypothesis that loss of β-TrCP expression at the mRNA level plays a role in enhancing REST function by reducing the levels of REST ubiquitination and degradation.

**Figure 7:**
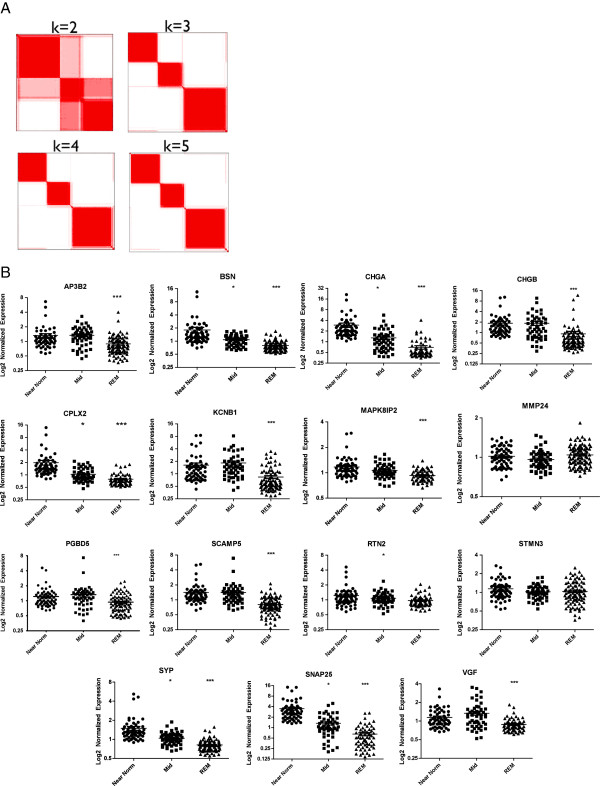
**Consensus clustering identifies 3 distinct REST subgroups.** Consensus clustering performed 1,000 iterations of delimiting TCGA tumors (X and Y axes) into 2, 3, 4 and 5 subtypes along a spectrum of REST function. (**A**) Dark red pixels represent a tumor that is consistently placed in the same group throughout the 1,000 iterations, while lighter pink pixels represent inconsistent cluster placement for tumors. (**B**) Expression of REST signature genes is significantly lower in the mid-range and REM tumor groups. *p<0.05 when compared to near-normal tumors, **p<0.05 compared to mid-range tumors, ***p<0.05 compared to both near-normal and mid-range tumors. Statistical analysis performed using one-way ANOVA Kruskal-Wallice test and Dunn’s multiple comparisons test in Graphpad Prism 5.01.

**Figure 8:**
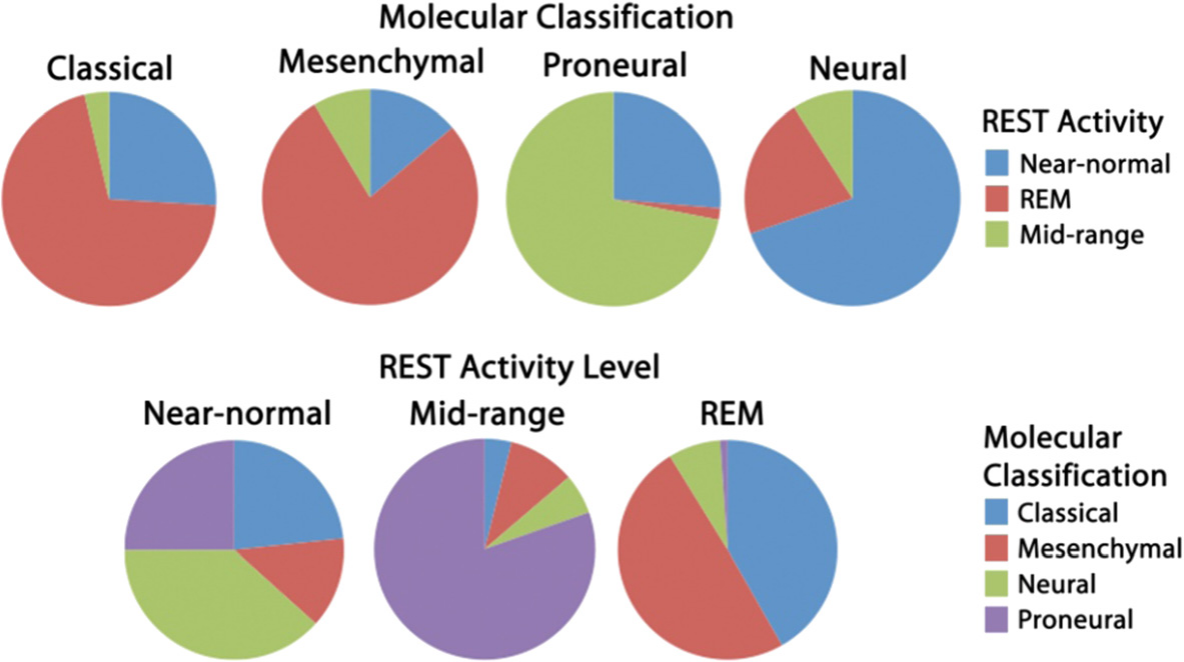
**Glioma subtype breakdown according to molecular classifications.** Tumors from a TCGA-unified dataset delimited by molecular subtypes and REST activity levels were tested for any overlap. Tumors with a mid-range REST activity level are predominantly proneural, while classical and mesenchymal gliomas subtypes are classified mostly as REST enhanced malignancies.

Despite their universally poor prognosis, glioblastoma multiformae tumors are quite heterogeneous at the molecular level. Recently, the array of recognized molecular subtypes of GBM has expanded to include neural, proneural, mesenchymal, and classical subtypes [21]. These subtypes represent not only a different gene expression profile, but also unique responses to treatment and a subtype specific enrichment in mutations and copy number aberrations. We next sought to determine if any of these established molecular subtypes segregated with the enhanced REST function observed in a subset of gliomas. In order to compare the overlap between the groups of molecular subtypes, we first had to divide the tumors into robust and reproducible groups delimited by REST function. To accomplish this, we made use of consensus clustering, an objective method for dividing tumors into reproducible subgroups according to their expression of a geneset. To divide outcome-associated tumors from the cancer genome atlas database along the lines of functional REST levels we applied consensus clustering to the REST gene signature, and 379 other genes that showed a very tight correlation (p<10^-8^, Pavlidis Template Matching) with REST function [22]. Using this approach, we determined that the TCGA GBM dataset was best divided into three robust and reproducible groups (Figure 7A). These 3 groups displayed different levels of REST target gene expression and could thus be divided into REST enhanced malignancies (REM), tumors with near-normal expression of REST targets (Near-normal), and tumors with mid-range REST target gene expression (Mid-range) (Figure 7B). When we compared tumor classifications we found that each of the REST-defined subtypes was comprised of a heterogeneous mixture of classical, mesenchymal, neural and proneural subtypes. Correspondingly, each of the four GBM subtypes was comprised of a heterogeneous mix of the three REST subtypes (Figure 8). These results suggest that these tumor groups represent distinct clusters of molecular subtypes, each with their own unique gene expression pattern.

**Figure 9:**
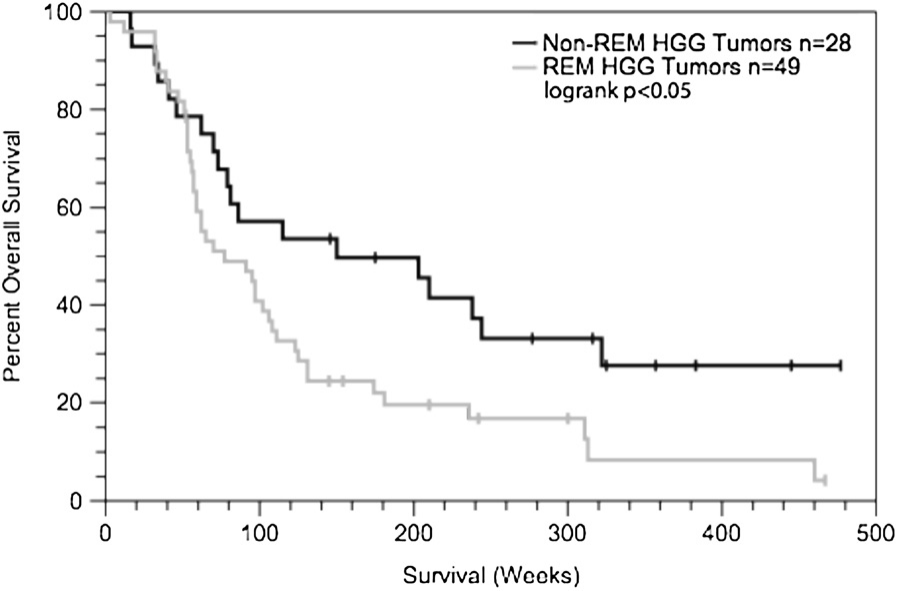
**REM gliomas are more aggressive than their near-normal counterparts.** REM tumors from high-grade glioma outcome-associated dataset GSE4271 are significantly more aggressive than their non-REM counterparts (logrank p<0.05). Median survivals for patients with REM (n=49) and non-REM tumors (n=29) were 77 weeks and 148 weeks, respectively. Mid-range (n=23) and near-normal (n=5) tumor groups were combined to add weight to the statistical analysis.

The role for molecular subtypes in cancer research has been expanding recently from helping researchers uncover molecular mechanisms of disease to aiding clinicians and patients in predicting disease course and response to treatment [23,24]. We sought to determine whether heightened REST function would similarly result in increased disease aggression [25]. To accomplish this, we assessed REST status in an outcome-associated dataset of high-grade gliomas (GSE4271 - grades III and IV) [26]. Upon stratification of the tumors into REM, near-normal, and mid-range REST functional groups, the patients with REM tumors showed a significantly more aggressive disease course than patients with non-REM tumors (Figure 9). These data suggest that increased REST function may be associated with more aggressive disease and coincide with mouse xenograft data showing reduced survival of mice injected with “High REST” glioma cells versus “Low REST” glioma cells [12].

**Figure 10:**
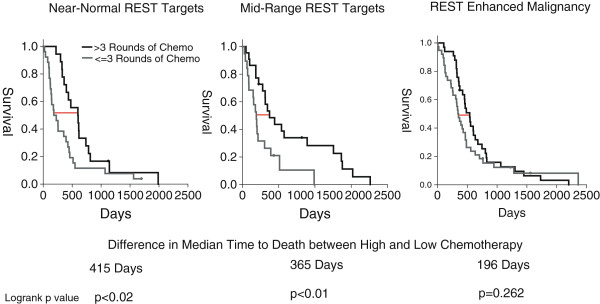
**Tumors with Near-normal and mid-range REST activity levels, show a significant survival benefit from additional chemotherapy.** GBM tumors from the TCGA dataset are associated with both outcome and treatment information. Here, we see that additional chemotherapy (4 or more cycles) is associated with significantly increased survival in near-normal and mid-range REST level tumors, but not REM tumors (logrank p<0.02, p<0.01, p=0.262 respectively).

Though the most aggressive grade IV GBM tumors are near-universally lethal, individual patient response to treatment is quite varied, making the decision to undergo high-dose chemotherapy over low-dose chemotherapy a difficult one [21]. To determine the susceptibility of each REST tumor subtype to high-dose chemotherapy, we compared outcome and treatment profiles for 200 tumors from the TCGA dataset. For patients with tumors expressing REST target genes at near-normal and mid-range levels, the administration of four or more rounds of chemotherapy is associated with a statistically significant increase in disease free survival over patients who underwent three or fewer doses of chemotherapy (Figure 10, logrank p<0.02, p<0.01, respectively). For patients with REM tumors, however, the increase in survival time associated with high-dose chemotherapy was not statistically significant (p=0.262).

To uncover possible mechanisms behind the differential disease course and response to treatment observed in REM tumors we searched for glioma-associated tumor suppressor genes whose mRNA expression levels co-varied with REST signature genes (Table 1). Of the identified glioma tumor suppressor genes, four had conserved REST binding sites (RE1 sites): neurofibromin 1 (NF1), brain expressed X-linked 1 (BEX1), cyclin dependent kinase inhibitor 1B (p27KIP1) and miR-124. NF1 is a Ras GTPase activating protein and its function is known to be lost in gliomas through mutation or degradation [28,29]. Recently published ChIP-ChIP data examining REST bound genes in glial cells found that REST directly binds NF1 endogenously in mouse oligodendrocytes, suggesting that NF1 is a direct target of REST repression [17]. Our work suggests that aberrant repression by REST may be another route to loss of NF1 in gliomas.

**Table 1:** Glioma tumor suppressors [Roopra]

**Gene symbol**	**Correlation**	**RE1 Site**	**ChiP**	**Summary**
NF1	p<0.000005	Intronic-TCTAGAACCAAGGCCAG	Abrajano	RasGAP tumorsuppressor. Loss of NF1 by mutation or degradation occurs in many gliomas and is associated with chemotherapy resistance
BEX1	p<10^-16	Intronic-TCTAGAACCAAGGCCAG	Johnson	Suppresses growth of glioma xenografts, reintroduction of BEX1 in glioma cell lines induces chemotherapy sensitivity
CDKN1B/p27KIP1	p<10^-9	Upstream-TCTAGAACCAAGGCCAG	-	Glioma tumor suppressor known to regulate cell growth, apaptosis and sensitivity to chemotherapy
mIR-124	p<10^-29	Multiple	Conaco	Glioma tumor suppressor that regulates proliferation and differentiation

REST ChIP Data are cited from either Abrajano et al [(2009),] Johnson et al [(2007)] or Conaro *et al (2006) *[[27]].

BEX1 is a glioma tumor suppressor gene, the overexpression of which effectively suppresses human glioma xenograft tumor growth in nude mice [30]. BEX1 mRNA expression is lost many gliomas, in part through promoter methylation [30]. Published ChIP-Seq analysis for REST bound genes found that REST directly binds the BEX1 gene in Jurkat T-cells, suggesting that it too is an endogenous REST target [3]. BEX1 mRNA shows a strong correlation with REST signature gene expression (p<10^-16^) and is two-fold lower in REM tumors than near-normal tumors, suggesting that the reduced BEX1 expression observed in these tumors may be due to increased REST function.

p27^KIP1^ is a cyclin dependent kinase inhibitor that regulates the G1/S transition by inhibiting a number of CDK complexes, including CDK2 and CDK4 [31]. Decreased expression of p27^KIP1^ in astrocytomas is associated with increased proliferation, and decreased patient survival [31,32]. p27^KIP1^ mRNA levels in tumors correlate with REST signature gene expression (p<10^-9^) and its gene contains a consensus REST binding site, suggesting that the reduced p27^KIP1^ expression observed in these tumors may be due to increased REST function.

Interestingly, loss of NF1, p27^KIP1^ and BEX1 are all associated with glioma chemotherapy resistance [30,31,33,34], suggesting that these genes may play a role in the reduced benefit of high dose chemotherapy in patients with REM tumors.

Here, we have provided evidence that REST function is increased in glioma tumors and that this heightened activity correlates with differential tumor aggressiveness and response to treatment. Our findings suggest a mechanism by which REST function may be enhanced in gliomas via loss of β-TrCP expression.

Importantly, we show that a decrease in a specific suite of REST target genes correlates with failure to respond to multiple round of chemotherapy, a finding of significant clinical impact.

## Methods

### Transcriptional analysis

Transcriptional analyses on the microarray data were performed using BRB-ArrayTools v3.7 (developed by Dr. Richard Simon and BRB-ArrayTools Development Team) and MultiExperiment Viewer 4.5.1. Tumor gene expression data were obtained from the NCBI Gene Expression Omnibus, and are identified by their GEO dataset record number, with the exception of the cancer genome atlas (TCGA) dataset, which was not available on GEO at the time of manuscript submission. TCGA datasets are described [21]. Hierarchical clustering was performed using a one-minus correlation metric with average linkage over centered genes. Cluster diagrams were produced using BRB Arraytools, Cluster 3.0 and TreeView software.

### Consensus clustering

The consensus clustering method was used to determine how many REST-activity delimited glioma subgroups may be reproducibly established in an unbiased fashion. First, genes that showed a high correlation of expression with the REST 24-gene signature at p<10^-8^ were defined using Pavlidis Template Matching using the MultiExperiment Viewer platform using the 200 tumor TCGA dataset. From this, 403 genes were identified and subjected to Consensus Clustering, which was performed using BRB array tools. One thousand iterations were used to classify tumors into 2, 3, 4 and 5 REST subtypes. In subsequent analyses, this analysis was used to classify tumors into just 3 REST-activity based subtypes.

### Gene set enrichment analysis

Gene Set Enrichment Analysis (GSEA) was performed using the GSEA program provided by the Broad Institute. The list of genes identified as likely REST targets were identified in Johnson *et al.* using ChIP-Seq with an anti-REST antibody. Genes were determined to be likely REST targets based on their ChIP-Seq enrichment in two independent experiments in a region carrying an RE1 site with a p-value of < 10^-4^.

### Kaplan-Meier analysis

Patient survival curves were generated using PRISM and MSTAT software (http://www.mcardle.wisc.edu/mstat/).

### Molecular classification comparison

Molecular classification of glioma tumors into classical, mesenchymal, proneural and neural subtype information for the TCGA tumor samples was published in Verhaak et al [2010]. To determine if tumor stratification by REST activity level overlapped significantly with established molecular classifications, these same tumors were re-classified using the consensus clustering method described above and co-incidence of classification is indicated both with respect to published molecular subtype (top) and REST activity level (bottom).

### Copy number analysis

Copy number analysis was performed using integrative genomics viewer from the Broad Institute (IGV - http://www.broadinstitute.org/igv/home). IGV was used to assess copy number variations in 141 glioma tumors in dataser GSE9635 previously published and characterized [18].

## Competing interests

The authors declare no conflict of interest.

## Authors’ contributions

MPW performed the analyses in Figures 1,4-10 and AR performed the GSEA analyses in Figures 2 and 3. Both authors read and approved the final manuscript.
